# Chronic Kidney Disease Is Characterized by “Double Trouble” Higher Pulse Pressure plus Night-Time Systolic Blood Pressure and More Severe Cardiac Damage

**DOI:** 10.1371/journal.pone.0086155

**Published:** 2014-01-23

**Authors:** Massimiliano Fedecostante, Francesco Spannella, Giovanna Cola, Emma Espinosa, Paolo Dessì-Fulgheri, Riccardo Sarzani

**Affiliations:** 1 Internal Medicine and Geriatrics and “Hypertension Excellence Centre” of the European Society of Hypertension, Department of Clinical and Molecular Sciences, University “Politecnica delle Marche”, Italian National Research Centre on Aging “U. Sestilli”, IRCCS-INRCA, Ancona, Italy; 2 Cardiology Clinic, Department of Cardiovascular Sciences, University “Politecnica delle Marche”, “Ospedali Riuniti”, Ancona, Italy; National Institutes of Health, United States of America

## Abstract

**Background:**

Hypertension plays a key role in chronic kidney disease (CKD), but CKD itself affects the blood pressure (BP) profile. The aim of this study was to assess the association of BP profile with CKD and the presence of cardiac organ damage.

**Methods:**

We studied 1805 patients, referred to our Hypertension Centre, in whom ABPM, blood tests, and echocardiography were clinically indicated. The glomerular filtration rate was estimated (eGFR) using the MDRD equation and CKD was defined as eGFR<60 mL/min/1.73 m^2^. Cardiac organ damage was evaluated by echocardiography.

**Results:**

Among patients with CKD there were higher systolic blood pressure (SBP) during the night-time, greater prevalence of non-dippers (OR: 1.8) and increased pulse pressure (PP) during 24-hour period, daytime and night-time (all p<0.001). Patients with CKD had a greater LVM/h^2.7^ index, and a higher prevalence of left ventricular hypertrophy and diastolic dysfunction (all p<0.001). Nocturnal SBP and PP correlated more strongly with cardiac organ damage (p<0.001). Patients with CKD had a greater Treatment Intensity Score (p<0.001) in the absence of a significantly greater BP control.

**Conclusions:**

CKD patients have an altered night-time pressure profile and higher PP that translate into a more severe cardiac organ damage. In spite of a greater intensity of treatment in most patients with CKD, BP control was similar to patients without CKD. Our findings indicate the need of a better antihypertensive therapy in CKD, better selected drugs, dosages and posology to provide optimal coverage of 24 hours and night-time BP.

## Introduction

Chronic kidney disease (CKD) is a major health problem and its prevalence is increasing worldwide [1]. A GFR lower than 60 mL/min/1.73 m^2^ for 3 months indicates CKD, regardless of the presence or absence of kidney damage [2]. More than 10% of the adult population of U.S. has some degree of renal impairment and almost half of these subjects (5–6%) are affected by a significant reduction of renal function (GFR <60 ml/min/1.73 m^2^) [3]. Data available from many European Countries are similar regarding prevalence and incidence of CKD [4] whereas only limited data concerning the prevalence of CKD in Italy have been published [5].

High blood pressure (BP) can be either a cause or a consequence of CKD [2]. Considering also the role of BP in diabetes, high BP is believed to be the leading cause of end-stage renal disease (ESRD). Every year, high BP causes more than 25,000 new cases of kidney failure in the United States [6]. A recent study shows that even pre-hypertension is significantly associated with an increased risk of CKD and it can be considered one of the relevant causes of CKD in the general population because of its high frequency [7]. In any case, when CKD is established BP increases. The results of Modification of Diet in Renal Disease (MDRD) study confirmed previous reports indicating that, in renal disease, hypertension is determined by the level of renal function [8].

Moreover, CKD is strongly associated with inadequate BP control. Many individuals with CKD are hypertensive and receive medications for controlling BP. However, the majority of these patients seem to be undertreated. Optimizing blood pressure treatment in this high-risk population can have a great potential in decreasing renal disease incidence and cardiovascular mortality [9].

Several studies reported that patients with an altered circadian blood pressure pattern, as non-dippers, have a higher risk of major cardiovascular events compared with patients with a normal pressure pattern [10]–[13].

Patients with CKD may have a non-dipping BP pattern. The explanation for this is not clear, but many factors have been proposed (e.g. defective natriuresis during daytime and increased blood pressure/natriuresis during the night) [14], [15].

To date, to the best of our knowledge, there is little data available, especially for the Italian population, regarding the inter-relationships between estimated glomerular filtration rate (eGFR), BP values and patterns (dipper, non-dipper) and echocardiographic parameters. Therefore, the rationale of our study was to investigate the associations between eGFR, BP values and patterns and echocardiographic parameters, to see how CKD and BP values and patterns may correlate with the presence of a cardiac organ damage.

The main aim of our study was to assess 24-h BP values and patterns and their relationship with eGFR and cardiac damage, in a population referred to a single “Hypertension Excellence Centre” for BP evaluation, in the hope to improve the clinical management of these patients in the absence of similar published data.

## Materials and Methods

All participants have given their informed written consent and clinical investigations have been conducted according to the principles expressed in the Declaration of Helsinki. This study was approved by the local institutional ethics committee (Comitato di Bioetica, Ospedali Riuniti, Ancona). We studied outpatients referred to our Hypertension Centre for BP related problems Inclusion criteria were: clinical indication to undergo ABPM, plasma creatinine measurement and echocardiography. Main exclusion criteria were: age<18 years, low-quality of pressure monitoring (rate of artifacts >25% and/or recording duration less than 20 continuous hours and/or <2 record per hour at daytime and 1 record per hour at night-time), and unreliable data regarding anti-hypertensive treatment. After exclusions, 1805 patients were studied; eGFR was estimated using the MDRD study equation [8] taking into account the dosage of creatinine by the Jaffé reaction.

CKD was defined as the presence of confirmed eGFR <60 ml/min/1.73 m^2^ in patients having the clinical indication to at least two creatinine determinations, the second one obtained 3 to 6 months after the first one (n = 962, 53.5% of the studied population, 100% of patients with eGFR <60 ml/min/1.73 m^2^). No significant differences in creatinine/eGFR were found between the two time points (assessed by paired t-Test), supporting the notion that 15% of our patients truly had CKD.

CKD stages were defined according to the international classification of National Kidney Foundation [2]. For each subject 24-h BP, daytime BP (defined as the BP values from 6 a.m. to 10 p.m.), night-time BP (defined as the BP values from 10 p.m. to 6 a.m) and pulse pressure (PP) [defined as systolic blood pressure (SBP) - diastolic blood pressure (DBP)] were evaluated. Daytime and night-time periods were defined based on a questionnaire, in which patients were asked about their sleeping behavior. Among patients under anti-hypertensive treatment, those with mean 24-h BP <130/80 mmHg, mean daytime BP <135/85 mmHg, and mean night-time BP <120/70 mmHg were defined as controlled hypertensive. Among untreated patients we labeled not-hypertensive those patients with mean 24-h BP <130/80 mmHg, mean daytime BP <135/85 mmHg, and mean night-time BP <120/70 mmHg [16]. We considered dippers those patients with a change in mean awake SBP to sleep SBP equal to or grater than 10%. We considered as night-to-day ratio the ratio between medium night-time and daytime values recorded performing ABPM. Night-to-day ratios were multiplied by 100, expressing night-time BP as a percentage of a daytime level. A ratio of 100% or higher indicated the absence of a BP fall at night [17]. After evaluating drug therapy adherence by MMAS [18] a treatment intensity score (TIS) was calculated to compare many different drug associations. As previously reported [19], the recorded daily dose taken by the patients was divided by the maximum recommended daily dose to obtain a proportional dose (called “intensity”) for that medication. The maximum recommended daily doses set by the Italian National Drug Agency (AIFA) were used for calculations. For completeness, dual-class drugs were separated into their components, and intensity was calculated separately for each chemical compound. The sum of all the different values was recorded as the TIS.

### Statistical analysis

Data were analyzed with the Statistical Package for Social Science version 13 (SPSS Inc. Chicago, Illinois, USA). A value of p<0.05 was defined as statistically significant. Quantitative variables were checked for normality. Normally distributed continuous variables were described as a mean ± standard deviation (SD). The unpaired t-test and ANOVA (for multiple comparisons) were used to compare normally distributed quantitative variables with Bonferroni correction for multiple comparisons. The χ^2^ test was used to analyze the differences between the categorical variables. Logistic and linear regression analysis and ANCOVA were used to create adjusted models.

## Results

General characteristics of the population with reliable ABPM and drug therapy are shown in Table 1. We studied 1805 patients: 1012 men (56.1%) and 793 women (43.9%). There were 1535 (85.0%) patients with eGFR ≥60 ml/min/1.73 m^2^ and 270 (15.0%) CKD patients. Hypertension was present in 1680 (93.1%) patients; 1194 (71.1%) were on drug treatment: 311 (26.0%) were controlled, 883 (74.0%) were not controlled. CKD stages are shown in Table 2. Only 10 patients (3.7%) of CKD subgroup had stages 4–5 (eGFR <30 ml/min/1.73 m^2^). Among the CKD patients 264 (almost all: 97.8%) were hypertensives, 218 (82.6%) were currently drug-treated, but only 63 (28.9%) had controlled BP.

**Table 1:** General characteristics of 1805 patients studied with ABPM, echocardiography, and eGFR.

	eGFR ≥ 60 ml/min/1.73 m^2^	CKD	P
Age (years ± SD)	53.6±13.2	66.2±11.4	**<0.001**
BMI (Kg/m^2^ ± SD)	27.4±4.4	27.7±4.7	n.s.
eGFR	79.0±12.3	51.2±8.9	**<0.001**

**Table 2:** Prevalence of CKD stages.

eGFR (ml/min/1.73 m^2^)	Mean ± Sd.	95% CI	n°	%
45≤eGFR<60	54.9±4.2	54.4–55.5	215	79.6%
30≤eGFR<45	39.3±3.7	38.1–40.4	45	16.7%
eGFR<30	24.2±5.3	20.4–28.1	10	3.7%

### CKD and BP values

**Figure 1:**
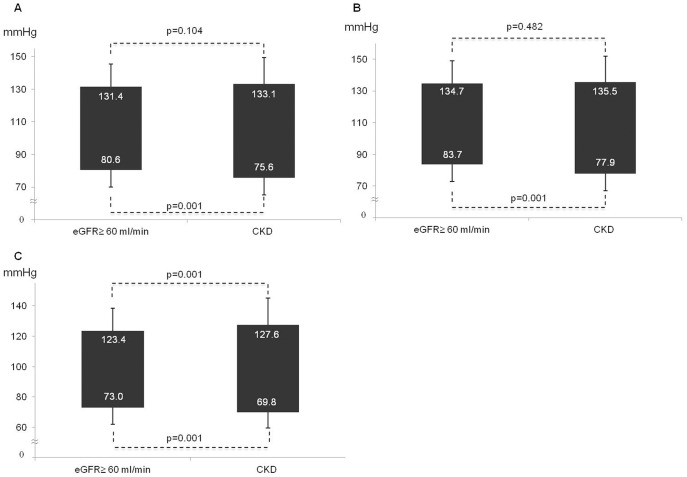
ABPM values in CKD patients vs. the rest of the population. Panel A. Difference in 24-h BP between patients with CKD and the rest of the population. Panel B. Difference in daytime BP between patients with CKD and the rest of the population. Panel C. Difference in night-time BP between patients with CKD and the rest of the population.

**Figure 2:**
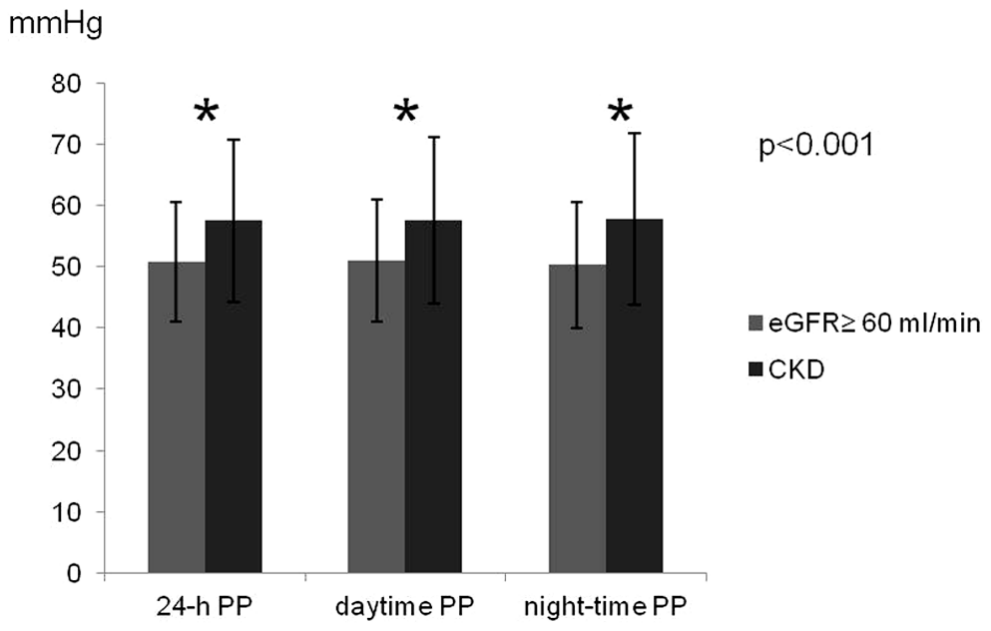
Difference in PP between patients with CKD and the rest of the population.

**Figure 3:**
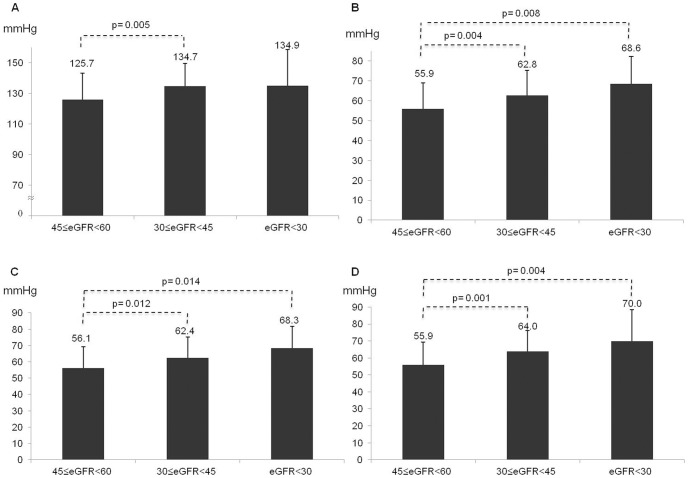
Correlation between ABPM values and CKD stages. Panel A. Difference in night-time BP between CKD stages. Panel B. Difference in 24 h PP between CKD stages. Panel C. Difference in daytime PP between CKD stages. Panel D. Difference in night-time PP between CKD stages.

As shown in Figure 1. Panel A-Panel B, 24-h and daytime SBP did not differ significantly between patients with or without CKD, but CKD patients had higher night-time SBP (127.6±17.6 vs. 123.4±15.2 mmHg; p<0.001; Figure 1. Panel C). On the contrary, lower 24 hours (75.6±10.4 vs. 80.6±10.6 mmHg; Figure 1. Panel A), daytime (77.9±10.8 vs. 83.7±10.9 mmHg; Figure 1. Panel B), and night-time (69.8±10.3 vs. 73.0±10.9 mmHg; Figure 1. Panel C) DBP were found in CKD where PP was higher in 24 hours (57.5±13.3 vs. 50.8±9.8 mmHg), daytime (57.6±13.5 vs. 51.0±10.0 mmHg) and night-time (57.7±14.0 vs. 50.3±10.3 mmHg) periods (all p<0.001) (Figure 2). These results were confirmed within worsening CKD stages (Figure 3 panel A–D), after adjustment for age and TIS, and in the linear regression model (Table 3): a significant relationship between the 24-h pressure profile and CKD was found only for nocturnal pressure values and for daytime, night-time and 24-h PP.

**Table 3:** Correlation between ABPM parameters and eGFR assessed by linear regression.

	B	P
**24-h SBP**	−0.019	0.413
**24-h DBP**	0.172	**<0.001**
**Daytime SBP**	0.007	0.765
**Daytime DBP**	0.192	**<0.001**
**Night-time SBP**	−0.066	**0.005**
**Night-time DBP**	0.118	**<0.001**
**24-h PP**	−0.197	**<0.001**
**Daytime PP**	−0.186	**<0.001**
**Night-time PP**	−0.205	**<0.001**

### CKD, dipping pattern, and night-to-day ratio

**Figure 4:**
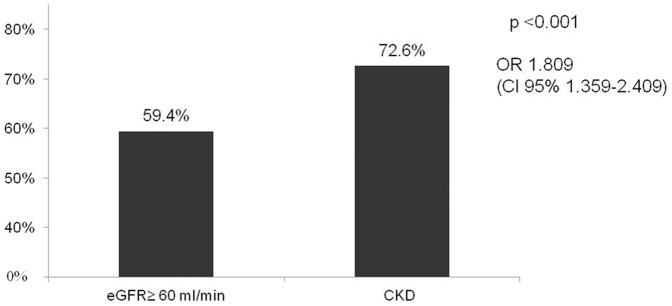
Prevalence of non dipping pattern in CKD.

Along with higher night-time SBP, patients with CKD showed higher prevalence of non-dipping pattern (72.6% vs. 59.4%; OR: 1.8, CI: 1.36–2.41; p<0.001) (Figure 4) and CKD was an independent risk factor for non- dipping pattern even after adjusting for sex, age, BMI, and TIS in a logistic regression model (Table 4). Patients with CKD also showed a linear association between eGFR reduction and night-to-day ratio (β = −0.123; p<0.001), another index of altered nocturnal BP.

**Table 4:** CKD is an indipendent risk factor for non dipping pattern after adjusting for sex, age, BMI, and TIS (logistic regression).

	OR	95% CI	P
**Sex (male vs female)**	0.855	0.659–1.109	n.s.
**Age**	1.013	1.002–1.023	**0.016**
**TIS** *	1.072	0.916–1.255	n.s.
**BMI**	1.055	1.024–1.087	**<0.001**
**CKD (CKD vs eGFR≥60)**	1.803	1.200–2.707	**0.005**

* TIS = Treatment Intensity Score.

### CKD, BP values and patterns and cardiac damage

**Figure 5:**
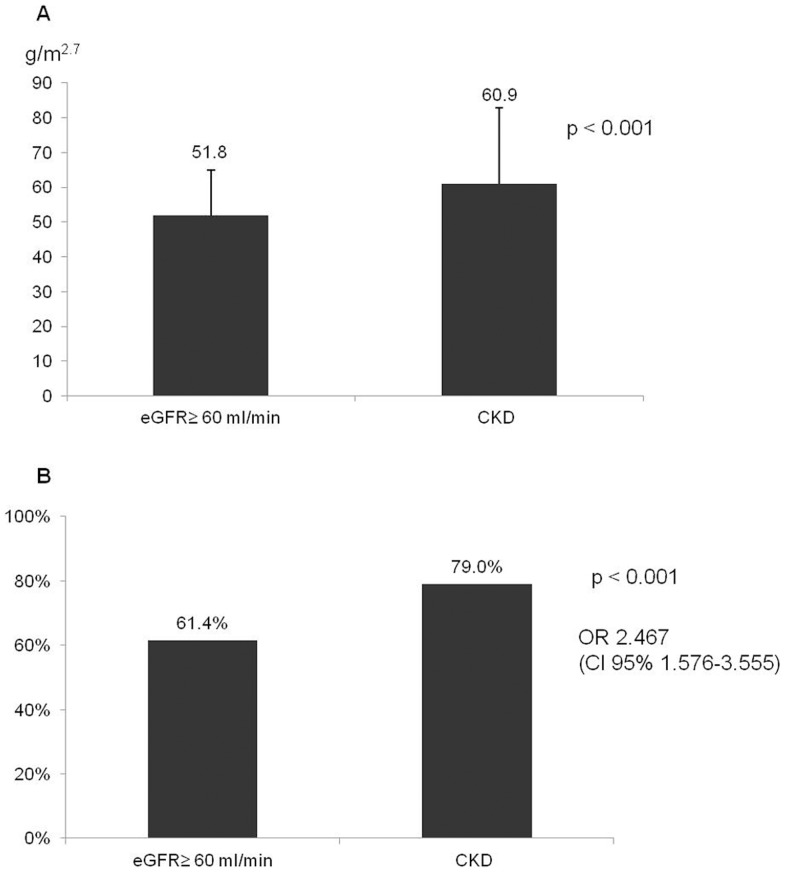
CKD and cardiac damage. Panel A. Difference in left ventricular mass/h^2.7^ between patients with CKD and the rest of the population. Panel B. Prevalence of ventricular hypertrophy in patients with CKD vs the rest of the population.

Considering the association with cardiac organ damage, patients with CKD had greater LVM/h^2.7^ (60.9±21.9 vs. 51.8±13.1 g/m^2.7^, p<0.001) and higher prevalence of left ventricular hypertrophy (79.0% vs. 61.4%, OR 2.48 CI:1.58–3.56; p<0.001) (Figure 5 Panel A–B). Indeed, linear regression model showed that night-time SBP as well as 24-h, daytime and night-time PP were independently associated with LVM/h^2.7^ after adjusting for age, sex, BMI, TIS and the presence/absence of CKD (Tables 5, 6, 7, 8). A higher prevalence of an index of diastolic dysfunction that was available in all patients, was also found in patients with CKD (E/A ratio <1: 79.3% vs. 54.5%, OR 3.2 CI:1.97–5.18; p<0.001).

**Table 5:** Night-time SBP associated with LVM/h^2.7^ in a linear regression model.

	B	p
**AGE**	**0.262**	**<0.001**
**SEX**	0.074	**0.005**
**CKD**	0.098	**<0.001**
**Night-time SBP**	**0.200**	**<0.001**
**TIS** *	0.092	**0.001**
**BMI**	**0.358**	**<0.001**

BMI and age were also two other important independent factors for LVM/h^2.7^.

* TIS = Treatment Intensity Score.

**Table 6:** 24-h PP associated with LVM/h^2.7^ in a linear regression model.

	B	p
**AGE**	**0.217**	**<0.001**
**SEX**	0.097	**<0.001**
**CKD**	0.097	**0.001**
**24-h PP**	**0.173**	**<0.001**
**TIS** *	0.082	**0.003**
**BMI**	**0.351**	**<0.001**

BMI and age were also two other important independent factors for LVM/h^2.7^.

* TIS = Treatment Intensity Score.

**Table 7:** Daytime PP associated with LVM/h^2.7^ in a linear regression model.

	B	p
**AGE**	**0.219**	**<0.001**
**SEX**	0.096	**<0.001**
**CKD**	0.099	**<0.001**
**Daytime PP**	**0.169**	**<0.001**
**TIS** *	0.082	**0.003**
**BMI**	**0.353**	**<0.001**

BMI and age were also two other important independent factors for LVM/h^2.7^.

* TIS = Treatment Intensity Score.

**Table 8:** Night-time PP associated with LVM/h^2.7^ in a linear regression model.

	B	p
**AGE**	**0.218**	**<0.001**
**SEX**	0.097	**0.001**
**CKD**	0.094	**0.001**
**Night-time PP**	**0.170**	**<0.001**
**TIS** *	0.084	**0.002**
**BMI**	**0.347**	**<0.001**

BMI and age were also two other important independent factors for LVM/h^2.7^.

* TIS = Treatment Intensity Score.

### CKD and BP control

Patients with CKD had a greater TIS (1.56±0.91 vs. 1.19±0.72; p<0.001) without significantly better BP control but, on the contrary, a tendency toward worse control (25.4% vs. 28.9%; p = n.s.). Among hypertensive patients treated but with BP not controlled, there was a significantly higher percentage (87.3%) of CKD with uncontrolled night-time SBP; OR = 2.4 (CI:1.11–5.33; p = 0.022). Isolated uncontrolled night-time SBP were also increased in CKD (prevalence 20.6%) vs patients with eGFR ≥ 60 ml/min/1.73 m^2^ (12.9%; p = 0.012) OR = 1.7 (CI:1.12–2.74).

## Discussion

We studied a large patient population with clinical indication to perform ABPM, lab test for plasma creatinine and echocardiography. The main findings in patients with CKD were an altered SBP profile at night-time (higher BP and a more frequent non dipper-pattern) and a higher PP which depends also on a significantly lower DBP. Higher SBP values in patients with CKD could be merely due to the higher prevalence of hypertension in these patients, as well as to a higher prevalence of severe hypertension in CKD. However, 24-hours and daytime pressures were not different between patients with and without CKD. Only night-time SBP and the dipper pattern were altered, leading to the hypothesis of a direct influence of CKD on nocturnal blood pressure profile. Impaired natriuresis in daytime may increase nocturnal SBP in order to compensate the diminished natriuresis with pressure natriuresis. Fujii et al [20] showed that the circadian rhythm of natriuresis is disturbed in patients with a non-dipper type of essential hypertension, in whom BP was usually sodium sensitive [21], [22]. Since the sodium sensitivity of blood pressure is considered to be caused by reduced GFR and/or increased tubular reabsorption of sodium [23], [24], such disarrangement may be clearly recognized in CKD in which renal function is deteriorated. In line with these findings, Fukuda et al [25] showed that, in the impaired renal function characterizing glomerulopathy, the nocturnal dipping of blood pressure is lost, resulting in enhanced urinary sodium and protein excretion during night-time. An important role seems to be played by the autonomic nervous system, since renal denervation has resulted in improvement of the nocturnal blood pressure profile in patients with moderate to severe CKD, leading to a reduction in night-to-day ratio and a restoration of the physiological circadian BP rhythm [26].

In our study, the association between non-dipping pattern and CKD was independent of age, sex, BMI and TIS, highlighting a direct influence of CKD on circadian pressure profile. The rise in differential pressures and the reduction in DBP were independent of age and TIS; in patients with CKD, this could be due to an increase in arterial stiffness. Increased atherosclerosis and arteriosclerosis, as well as the metabolic alteration present in CKD with secondary calcifications of the intima and media layers of the artery wall, are likely contributors to this process [27]. This is probably of great relevance in the population with CKD as the patients with CKD are likely to be older (in our study the medium age of CKD patients was 66.2±11.4). Franklin et al [28] demonstrated that, from age 60 and above, DBP was negatively related to coronary heart disease (CHD) risk, when considered together with SBP, and PP emerged as the best predictor. The increased CHD risk due to isolated systolic hypertension may depend not only on the elevated peak of SBP reached in the aorta (ie, increased afterload) but also on the low DBP (ie, potentially favoring a decreased coronary blood flow) [29]. In patients older than 60, with central and peripheral PPs approximating each other, PP becomes the dominant predictor of CHD risk, incorporating both the positive predictive role of SBP and the negative predictive role of DBP. In this way CKD worsens the age-related arterial stiffness and potentiates its effects. Our data overall strongly support the concept that once CKD appears and worsens, some aspects of blood pressure worsen too, as a consequence of CKD implications. These BP aspects (higher night-time BP, more non-dippers and higher PP) are known as important cardiovascular risk factors [30]–[32] and they translate in greater severity of cardiac damage in CKD.

Our patients with CKD had a higher prevalence of both myocardial hypertrophy and diastolic dysfunction, suggesting that the higher PP and the night-time SBP significantly increased the heart load. Linear regression analysis revealed a strong association between nocturnal blood pressure, PP and cardiac organ damage. CKD itself was also independently associated with cardiac damage, suggesting that other factors (such as CKD-related metabolic alterations) in addition to BP are involved in the higher prevalence of cardiac damage and cardiovascular risk in these patients. Ad-hoc designed studies will be needed to investigate the association between metabolic alterations in CKD and cardiac damage, relevant aspects that were not the focus of our present work. Despite greater intensity of treatment in CKD, BP control is similar in the overall distribution. However, during night-time, there is a higher percentage of subjects with poor nocturnal control in CKD, a finding enlightened only by ABPM. Because of the lack of recognized pressure target for ABPM, we had to use arbitrary cut offs to define BP control, referring to the values in the range of normotension for ABPM in general population. Notwithstanding, according to initial evidences [33], ABPM target pressure values in patients with CKD should be lower than the one we considered.

## Conclusions

Our “real-life patient” data are relevant for clinicians that often evaluate BP-related clinical problems on the basis of a single plasma creatinine value in the initial evaluation of outpatients. Our study shows that patients with a reduced eGFR (<60 ml/min/1.73 m^2^) had an altered 24-hour BP profile, especially at night-time. Higher night-time SBP, a more frequent non-dipper pattern and higher PP are related with more severe cardiac organ damage.

Hypertension is the main cause of CKD and an accurate measurement of BP is the critical first step for the proper management of hypertensive patients. Our data highlight the fundamental role of ABPM in optimizing antihypertensive therapy when eGFR is low. The altered night-time BP pattern in CKD suggests the need of improving BP therapy in these subjects, by choosing drugs, dosages and timing to cover the 24 hour period, taking at least one drug at bedtime, and using effective diuretics. ABPM should be the preferred method in the clinical management of patients with CKD as well as in clinical studies.

### Study limits

The main limitation of our study is the use of post-hoc analysis of consecutive ABPM recordings that were performed for usual clinical reasons (including/excluding hypertension diagnosis, verify the BP control in treated hypertensive patients). Although a selection of patients was unavoidable (e.g. the three “inclusion criteria”: ABPM, creatinine and echocardiography), patient selection was based only on the best clinical practice for the management of arterial hypertension.

Overall, our analysis is based on one creatinine/eGFR and this is certainly a limitation. Nevertheless, our data fit in the context of current literature because most of the data available have been obtained with a similar approach. Our “real-life patient” data are relevant for clinicians that make their decisions most often on the basis of a single creatinine determination during the initial evaluation of “stable” outpatients coming for BP related problems. Moreover, patients having the clinical indication for at least two creatinine determinations, had the second one taken 3 to 6 months apart. There were no significant differences in creatinine values/eGFR between the two time points, supporting the notion that 15% of our patients truly had CKD.

Our study is based on “real-life” clinical practice, and we have not been able to include all potential causes of non-dipping pattern in the statistical adjustments (e.g. presence of obstructive sleep apnoea documented by cardio-pulmonary monitoring), a limitation similar to other studies that investigated the relationship between lower eGFR and ABPM patterns. Moreover, we didn't study some CKD biochemical factors (e.g. regarding Ca, P metabolism) and their role in influencing the association between CKD, BP profile and cardiac damage. This was because some metabolic parameters of CKD are not routinely assessed when patients are referred for BP evaluation in a cardiovascular setting. However, the overall night-time results (night-time SBP, night-time PP, non dipping pattern) and increased 24 h PP in our large population, even after statistical adjustments, strongly indicate an altered pattern of night-time BP in CKD patients, with a negative impact on the heart and therefore with important clinical implications.
